# A Simulation and an Experimental Study of Space Harpoon Low-Velocity Impact, Anchored Debris

**DOI:** 10.3390/ma15145041

**Published:** 2022-07-20

**Authors:** Wei Zhao, Zhaojun Pang, Zhen Zhao, Zhonghua Du, Weiliang Zhu

**Affiliations:** 1 School of Mechanical Engineering, Nanjing University of Science and Technology, Nanjing 210094, China; zhao0219@njust.edu.cn (W.Z.); duzhonghua1971@163.com (Z.D.); bywzwl@163.com (W.Z.); 2 Aerospace System Engineering Shanghai, Shanghai 201109, China; zhaozhen101@163.com

**Keywords:** space harpoon, impact, friction, embedding length, launch initial velocity

## Abstract

The space harpoon is a rigid-flexible, coupled debris capture method with a simple, reliable structure and a high adaptability to the target. For the process of impacting and embedding the harpoon into the target plate, the effect of friction at a low-velocity impact is studied, and the criteria for effective embedding of the harpoon and the corresponding launch velocity are determined. A simulation model of the dynamics of the harpoon and the target plate considering tangential friction is established, and the reliability of the numerical simulation model is verified by comparing the impact test, focusing on the kinetic energy change and embedding length during the impact of the harpoon. The results show that the frictional effect in the low-velocity impact is more obvious for the kinetic energy consumption of the harpoon itself, and the effective embedding of the harpoon into the anchored target ranges from 50~90 mm, corresponding to a theoretical launch initial velocity between 88.4~92.5 m/s.

## 1. Introduction

With the increasing frequency of space launch activities, the amount of space debris has dramatically increased [1]. According to data from the U.S. Space Surveillance Network (SSN) long-term monitoring of targets above 10 cm in low Earth orbit and targets above 1 m in geostationary orbit, it is known that the number of objects in Earth orbit has surged from approximately 11,000 in 2000 to nearly 20,000 in 2020 over the last two decades [2,3]. Currently, most policies, standards, and other regulations are reflected in curbing the increase, but the existing space debris in orbit still poses a threat to spacecraft operating in orbit. Therefore, active debris removal (ADR) has received more attention in recent years. Studies have shown that when five pieces of space debris are cleaned up each year, the probability of collision of debris and satellites can be effectively mitigated and reduced [4,5]. Space harpoon capture is a rigid-flexible, coupled debris capture method with a simple, reliable structure and a high adaptability to the target [6,7].

The satellite platform carried with the harpoon payload launches the harpoon by electromagnetic spring or pyrotechnics, which impacts the debris and anchors it to the target, where it is dragged through the atmosphere and burned up by a tow rope between the platform and the harpoon. Space debris is mostly non-cooperative targets. In order to solve the problem of difficult identification and capture caused by the complex attitude of non-cooperative targets and to achieve effective anchoring of the harpoon to the target, a higher launch velocity is usually required to increase the kinetic energy of the harpoon when it hits the target. Excessive launch velocity on the one hand will produce a large recoil force on the platform that will cause the platform to become unstable, on the other hand, the harpoon may penetrate the debris and anchor poorly. In order to reduce the impact on the platform during launch and to ensure that the harpoon impacts and anchors the debris effectively, the launch velocity must be controlled as much as possible. The problem of harpoon impact and anchoring of space debris can be considered as kinetic energy projectile penetration and embedding in thin plates.

The current research on the impact penetration problem still mainly concerns target plate failure and ballistic limiting velocity involved in different head shape projectiles hitting different types and thicknesses of targets vertically or inclined, and the velocity problem involved in low-velocity impact and anchored and embedded targets is less studied. Campbell et al. [8] analyzed the effect of mesh sensitivity on ballistic terminal velocity by comparing the ballistic terminal velocity problem of flat-headed and ogive harpoons impacting aluminum honeycomb panels through simulation and experiment; Dudziak et al. [9] studied the change in ballistic limiting velocity of blunt projectiles and conical projectiles impacting 3 mm steel plates, and they analyzed the cracking pattern of steel plates at low temperatures; Aglietti et al. [10] analyzed the velocity and the impact of harpoons during harpoon impact experiments on aluminum honeycomb panels in the RemoveDEBRIS project; Fras et al. [11], Wang et al. [12], Kpenyigba et al. [13], and Deng et al. [14] studied the ballistic terminal velocities and the target failure forms of three structures with blunt projectile, hemispherical projectile and ogival projectile impacting high-strength armor steel, 2024-T351 aluminium alloy plate, mild steel sheets, and 6061-T651 aluminium alloy thin plates by conducting simulations and experiments; Rusinek [15] analyzed the effect of bullet diameter on the ballistic terminal velocity under the same initial kinetic energy and the failure form of the target plate.

This paper compares the current situation at home and abroad, combines the designed harpoon structure, uses simulation and experimental verification methods to discuss the influence caused by the friction coefficient between the harpoon and the target plate at low-velocity impact, studies the relationship between the velocity and the embedding length when the harpoon impacts the 2A12 aluminum alloy plate under different conditions of initial velocity, and further obtains the launch velocity problem corresponding to the harpoon within the effective anchoring interval length.

## 2. Impact Experiment

**Figure 1:**
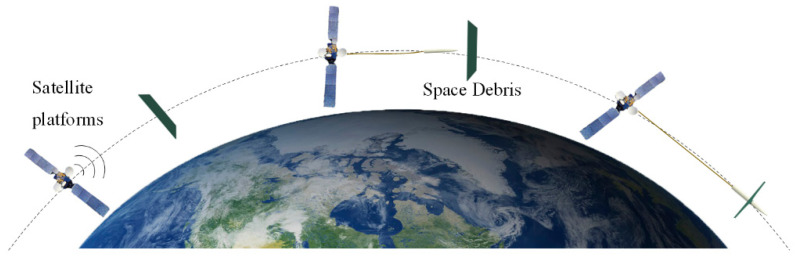
Work scene.

The working scene of harpoon capture is shown in Figure 1. A satellite platform detects the orbital position of the debris and approaches the target debris after maneuvering to change the orbit, and the platform launches the harpoon, which is connected to the platform by a high-strength tether. The harpoon impacts the debris and effectively embeds into the debris by the kinetic energy provided by the launch, after which the platform drags the debris down to burn. This paper focuses on the stage of harpoon impacting and embedding debris.

### 2.1. Harpoon and Target Plate Model

**Figure 2:**
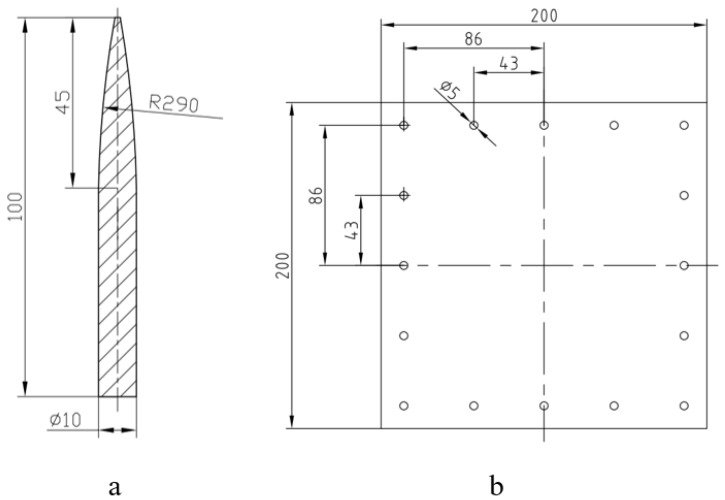
Structural model. (**a**) Harpoon, (**b**) Target plate.

The structure of the harpoon and the target plate used in the test is shown in Figure 2. The harpoon used in the figure is 100 mm in length and 10 mm in diameter—the head is the harpoon penetration part with an ovoid structure design—the length is 45 mm, and there is a columnar structure at the bottom. The material of the harpoon is S45C steel, with a HRC hardness between 55 and 58 after heat treatment. The total mass of the harpoon is 48.4 g. The target plate material in the simulation process is 2A12 aluminum alloy plate commonly used in spacecraft skin and other components, and the target plate is a homogeneous plate with a size of 200 mm × 200 mm and a thickness 2 mm, with 16 M5 bolt holes evenly distributed around.

**Figure 3:**
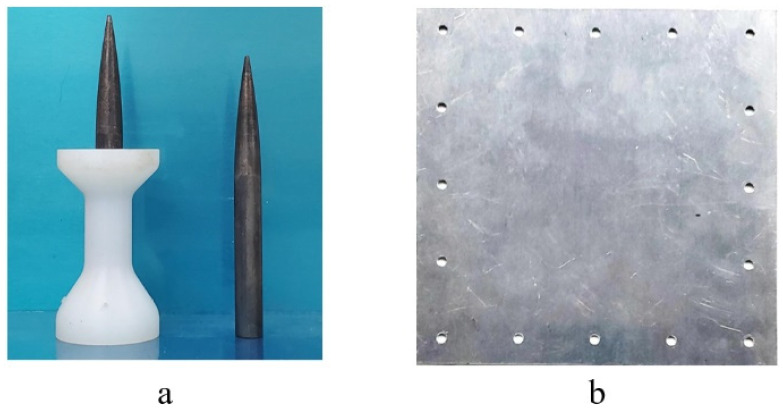
Harpoon and target plate physical drawing. (**a**) Harpoon, (**b**) Target plate.

The physical drawing of the harpoon is shown in Figure 3a, the white part is the nylon sabot, which is used as the support part to ensure the stability of the harpoon during launch, and the harpoon is embedded in the sabot when launch, and put into the air gun together with the sabot. The target plate is fixed to the target frame by 16 evenly distributed M5 bolts, which is used to ensure that the target plate will not be displaced axially and radially after being hit by the harpoon during the test, and its configuration is shown in Figure 3b.

### 2.2. Experimental Work

**Figure 4:**
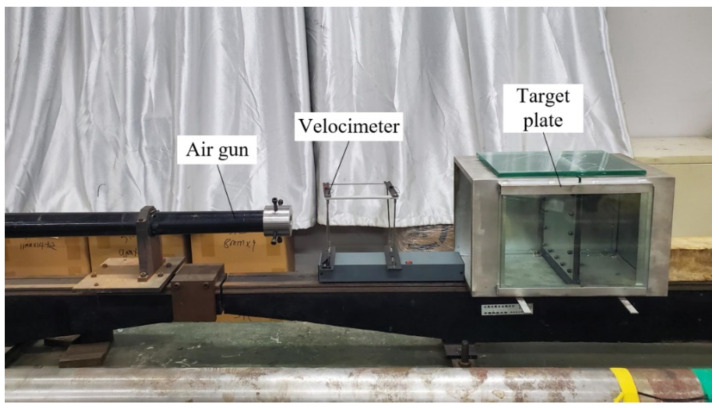
Impact experiment equipment.

Design the test plan shown in Figure 4, the test equipment mainly includes a gas chamber, 37 mm caliber air gun, velocimeter, target frame, etc. In the test, the velocity of the harpoon launch is changed by controlling the nitrogen pressure provided by the gas chamber, and the distance from the mouth of the air gun to the target plate is 1.2 m. There is a recoil sleeper fixed after the target frame to prevent the target plate from being impacted, which will drive the target frame to move back and affect the test results.

### 2.3. Experimental Results and Analysis

**Figure 5:**
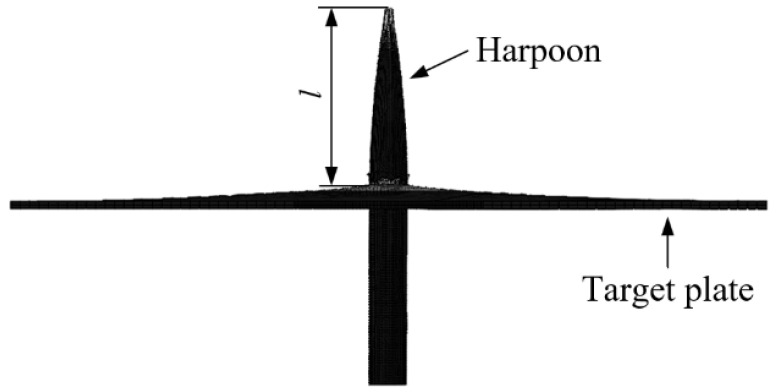
Maximum embedding length of harpoon.

**Figure 6:**
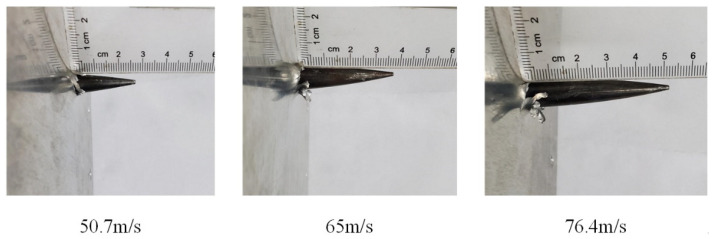
Experimental results.

The process of the harpoon impacting the target plate is mainly the harpoon pushing the target material through the “ductile hole expansion” mechanism, which makes the harpoon pass through, causing the thin plate near the impact point to be stretched and bent, and the phenomenon of petal cracking will occur under certain conditions. The distance from the tip of the harpoon embedded in the target plate to the maximum height of the projection is defined as the maximum embedding length, which is shown as the length of *l* in Figure 5. The test was conducted using the above test scheme, and the test results are shown in Figure 6. After the test, there was no obvious structural damage to the harpoon itself, but there were obvious scratches on the harpoon, which was caused by strong friction during the process of harpoon impact, and finally the harpoon was embedded in the target plate, and the harpoon was closely fitted to the target plate without obvious movement in the radial direction. The length of harpoon embedment was 22 mm, 37 mm, and 51.5 mm at an impact velocity of 50.7 m/s, 65 m/s, and 76.4 m/s, respectively.

## 3. Numerical Simulation

Based on the above test conditions and results, this paper uses ABAQUS/Explicit module to establish the dynamics simulation model of harpoon and target plate, and further uses the simulation test to study the relationship between velocity and friction coefficient on the length of the harpoon embedded in the target plate, and analyzes the results.

### 3.1. Harpoon–Target Plate Material Model and Equation of State

During the high-velocity impact, the harpoon and the target plate materials are subjected to large deformations and high strain rates, while the temperature of the material also dramatically increases. The Johnson–Cook constitutive model is well suited to characterize the dynamic mechanical behavior of materials under impact, in particular the characteristic behavior of the mechanical response of the material during intrusion, such as the adiabatic shear phenomena [16]. The model uses the von Mises yield surface and its flow law to take into account the strain, strain rate hardening, and temperature rise softening of the material, assuming that the isotropic strain, strain rate strengthening, and temperature rise softening factors of the material are decoupled. The Johnson–Cook material model was used for both the harpoon and the target plate in the simulation [17], and the expression of the Johnson–Cook material model is as follows.

(1) $$\sigma_{e}=\left(A+B\varepsilon_{e}^{n}\right)\left(1+C\ln{\dot{\varepsilon }}_{e}^{*}\right)\left(1-T^{*m}\right)$$

where $\sigma_{e}$ is the equivalent effect force; *A* is the yield strength of the material at the reference strain rate and reference temperature; *B* and *n* are strain strengthening coefficients; *C* is the strain rate sensitivity coefficient; $\varepsilon_{e}$ is the equivalent plastic strain; ${\dot{\varepsilon }}_{e}^{*}$ is the dimensionless equivalent plastic strain rate; ${\dot{\varepsilon }}_{e}^{*}={\dot{\varepsilon }}_{e}/{\dot{\varepsilon }}_{0}$, ${\dot{\varepsilon }}_{0}$ is the reference strain rate, usually takes the value of 1.0 s^−1^; T* is the dimensionless temperature, T* = (*T* − *T*_r_)/(*T*_m_ − *T*_r_), *T*_r_, *T*_m_ are room temperature and material melting points, respectively.

The strain at fracture is [12]:

(2) $$\varepsilon_{f}=\left[D_{1}+D_{2}\exp\left(D_{3}\sigma^{*}\right)\right]\left(1+D_{4}\ln{\dot{\varepsilon }}_{e}{}^{*}\right)\left(1+D_{5}T^{*}\right)$$

where *D*_1_~*D*_5_ are material parameters, $\sigma^{*}$ is the stress triaxiality, defined as $\sigma^{*}=\sigma_{m}/\sigma_{\mathrm{eq}}$, where $\sigma_{m}$ is the hydrostatic pressure and $\sigma_{\mathrm{eq}}$ is the von Mises equivalent force.

The Johnson–Cook failure model uses the theory of cumulative damage to consider the effects of stress states, strain rates, and temperature changes on material damage. It is considered that the damage does not affect the strength of the material and that the damage variable has an initial value of 0, when it reaches 1, the material fails. The damage evolution of a unit is defined as [18]:

(3) $$D=\sum \left(\Delta \varepsilon_{\mathrm{eq}}/\varepsilon_{f}\right)$$

where *D* is the damage parameter, $\varepsilon_{\mathrm{eq}}$ is the equivalent plastic strain increment during the integration cycle.

The parameters of the harpoon and the target plate materials are shown in Table 1, Reprinted with permission from Refs. [19,20].

### 3.2. Harpoon–Target Plate Simulation Model

**Figure 7:**
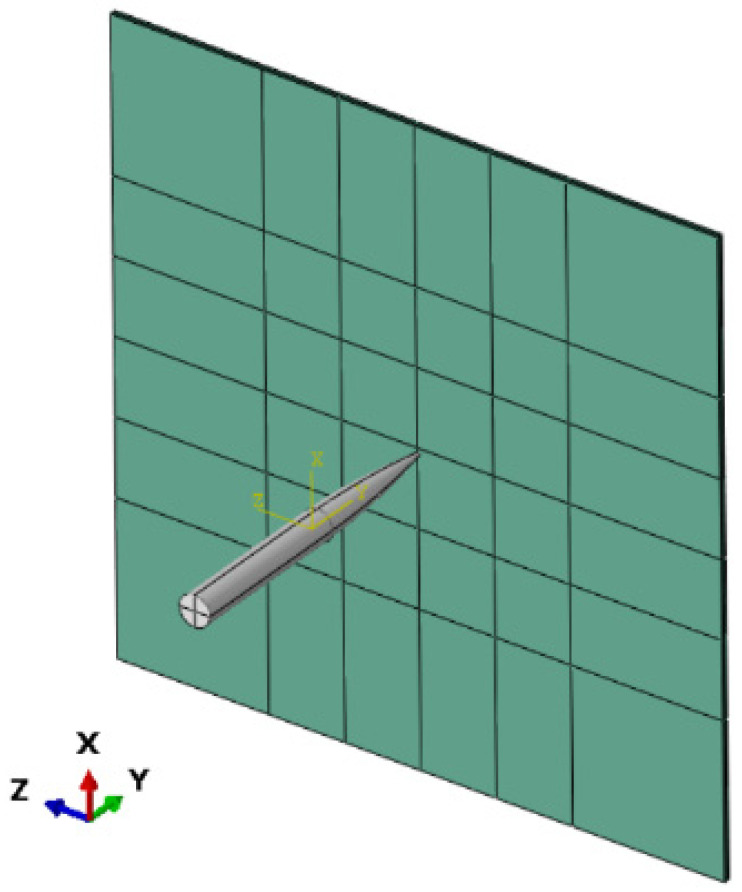
Finite element model.

ABAQUS/Explicit module is used to establish the finite element simulation model of harpoon impact, as shown in Figure 7. The model consists of two solid parts, harpoon and target plate, and the landing angle of the harpoon is 0° during the simulation. The contact between the harpoon and the target plate is modeled using the penalty method with a finite sliding formulation. The four boundaries of the target plate are set as fixed constraints, and the harpoon is simulated by setting different initial velocities in predefined fields to simulate the impact results.

**Figure 8:**
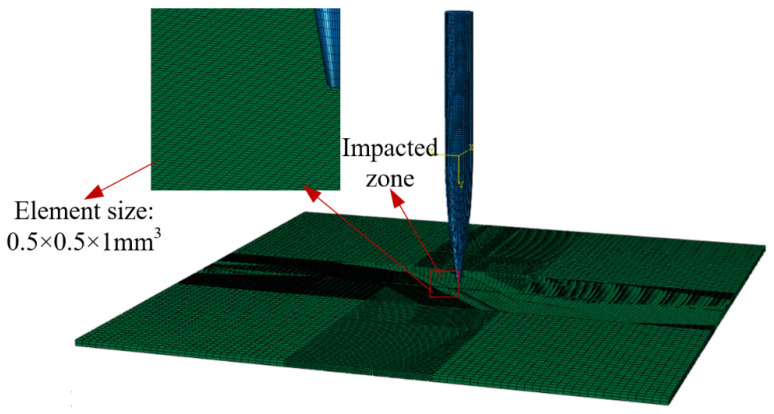
Mesh model.

Both the harpoon and the target plate use the structural mesh shown in Figure 8, and the mesh at the center of the target plate is encrypted to simulate the impact effect and to improve the computational efficiency. The harpoon is divided into 49,800 cells and the target plate cell number is 51,200. Meanwhile, in order to avoid distortion of the mesh during the simulation, which may affect the accuracy of the analysis, both mesh cell types are C3D8R (eight-node linear hexahedral cells) [21].

### 3.3. Comparison of Experimental and Simulation Conclusions

The accuracy of the simulation model and the material constitutive parameters is verified by comparing the above air gun experimental data through simulation, and the dynamic friction coefficient between the harpoon and the target plate is set in the simulation, and the simulation data are compared with the relevant data derived from the experiments in Section 2.3 of this paper, and the conclusions are shown in Table 2.

Comparing the simulated and the experimental data, the errors of the harpoon embedded length data obtained by the three groups of simulation and the experimental data are 5%, 4.3%, and 0.91% respectively, which are small and within the allowed range. The simulation results of the harpoon embedded length are in good agreement with the actual experimental results. It can be seen that the material model and the material-related parameters used in the simulation are reasonable.

### 3.4. The Effect of Friction on Harpoon Head Embedding

In order to study the influence of whether there is friction between the penetrating part of the harpoon head and the target plate on the embedded length, the simulation analysis of whether there is friction between the harpoon and the target plate is carried out, respectively. The results of the simulation with and without friction on the embedding length of the harpoon at different velocity are shown in Table 3.

**Figure 9:**
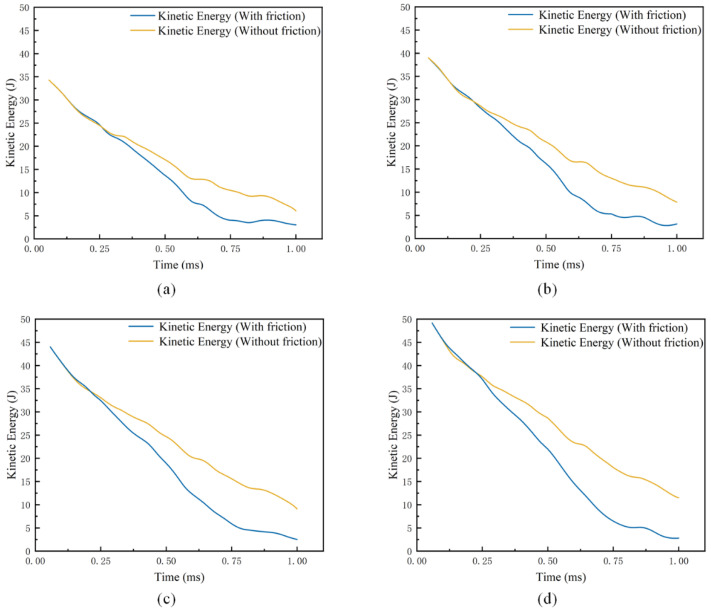
Kinetic energy evolution curve. (**a**) Evolution of kinetic energy at a velocity of 37.5 m/s; (**b**) Evolution of kinetic energy at a velocity of 40 m/s; (**c**) Evolution of kinetic energy at a velocity of 42.5 m/s; (**d**) Evolution of kinetic energy at a velocity of 45 m/s.

According to the simulation test results, under the simulation velocity conditions set above, the first 1 ms of the simulation is mainly for the impact of the harpoon head on the target plate. Using the kinetic energy data of the harpoon in the first 1 ms of time, the relationship change is shown in Figure 9.

As can be seen from Figure 9, the kinetic energy of the harpoon starts to decrease when the head of the harpoon touches and impacts the target plate in the initial 1 ms. With the increasing impact depth of the harpoon, under the simulation condition of adding the friction coefficient, the impact caused by the tangential contact friction between the harpoon and the target plate gradually becomes larger, the harpoon significantly loses kinetic energy, and the impact ability rapidly decreases. When the kinetic energy of the harpoon dropped to 4.5 J, the harpoon basically lost forward ability; the kinetic energy curve was affected by the fluctuation of the target plate, and it finally gradually decreased to 0.

**Figure 10:**
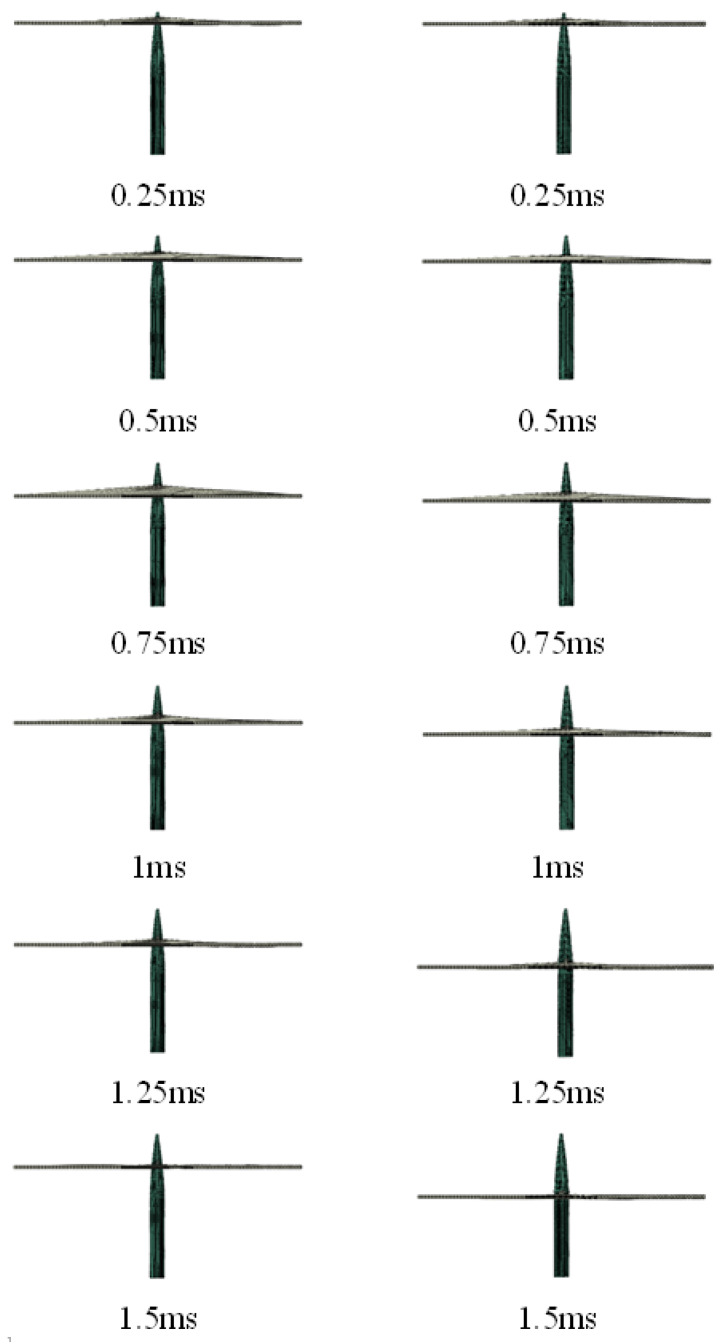
Variation of harpoon impact embedded length (left side is with friction coefficient, right side is without friction coefficient).

When the initial velocity of the harpoon was 50 m/s, the relationship between the length of the harpoon and the embedded length of the target plate with and without friction was obtained, as shown in Figure 10. At the initial stage of impact, the difference between the length of the embedded target plate with and without the friction coefficient is not obvious; with the depth of the harpoon embedded, when the impact time reaches 0.75 ms, the length of the harpoon embedded length is more and more affected by the tangential friction. At this point in the frictionless conditions, when the impact time reaches 1 ms, the remaining kinetic energy of the harpoon is 17.2 J, and the head of the harpoon can successfully complete the penetration of the target plate. Under the condition of friction coefficient, the kinetic energy of the harpoon gradually decreases; and, at 1 ms, the kinetic energy of the harpoon decreases to 4 J, which is only approximately 1/4 of the frictionless condition; at this time, the kinetic energy of the harpoon itself can no longer continue to penetrate the target plate, and under the effect of friction and fluctuation of the target plate, the kinetic energy of the impact of the harpoon is gradually eliminated until it decreases to 0. It can be seen that for the head penetration part of the harpoon, the tangential friction between the harpoon and the target plate has a greater impact on the impact efficiency; so, for this low-velocity impact problem, the impact of the tangential friction between the harpoon and the target plate needs to be considered in the simulation.

### 3.5. The Effect of Friction on Harpoon Column Surface Embedding

**Figure 11:**
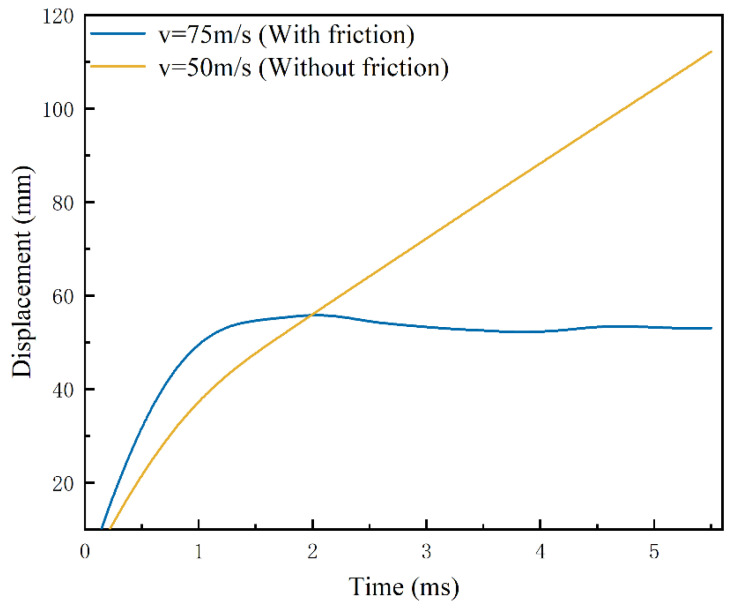
Harpoon displacement curve.

**Figure 12:**
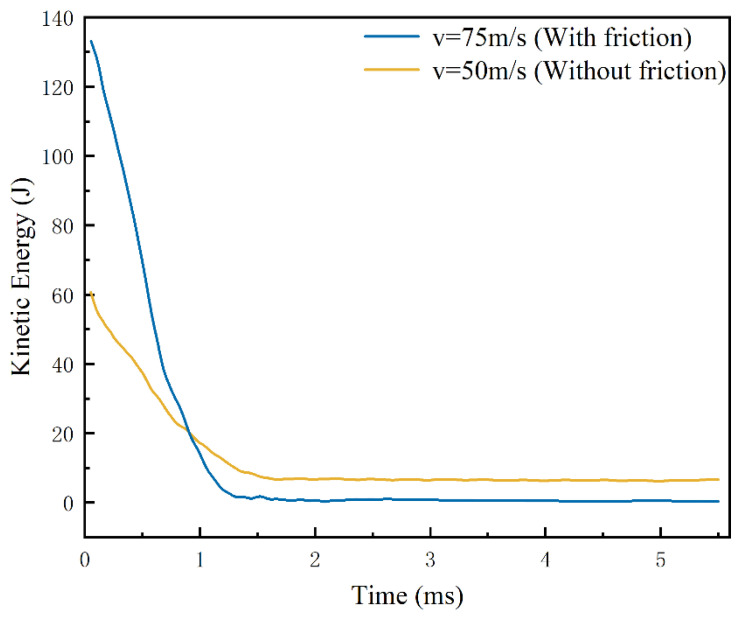
Kinetic energy curve.

When the head of the harpoon finishes impacting the target plate, the kinetic energy of the harpoon decreases and gradually loses forward ability, and it is embedded in the target plate due to the mutual friction coefficient and the fluctuation of the target plate deformation. Analysis of the above embedding data when the initial velocity of the harpoon is 50 m/s under frictionless conditions reveals that when the time is 1.5 ms, the embedding length of the harpoon is more than 45 mm; at this time the head of the harpoon has penetrated the target plate for the contact stage between the column surface part of the harpoon and the target plate. Additionally, when there is tangential friction between the harpoon and the target plate, the velocity needs to reach more than 70 m/s; after 1 ms, the harpoon penetration part can penetrate the target plate, and the displacement change curve of the harpoon is shown in Figure 11. A comparison of simulation results at a velocity of 75 m/s with friction conditions with the results at 50 m/s without friction conditions was made, the kinetic energy curve changes are shown in Figure 12.

From the data analysis of Figure 11 and Figure 12, the kinetic energy of the harpoon dropped to approximately 7.75 J after 1.5 ms under the frictionless condition; there was no obvious change in kinetic energy after 1.5 ms, and the kinetic energy of the harpoon was kept at approximately 6.5 J at all times until the harpoon flew completely through the target plate after 5 ms, and the harpoon was not effectively embedded in the target plate. In the case of friction, the impact velocity of 75 m/s after 1.1 ms of kinetic energy dropped to 7.74 J, after that the displacement of the harpoon column surface in the target plate change rate is relatively flat; the friction between the harpoon and the target plate consumes the kinetic energy of the harpoon itself, the kinetic energy of the harpoon in 1.1~1.25 ms rapidly declines, and it is finally embedded in the target plate. Due to the impact of the target plate fluctuations, the kinetic energy of the harpoon changes slightly and finally decreases to 0, as the fluctuation of the target plate stops.

It can be seen that when the harpoon deeply impacts the stage of contact with the target plate, the kinetic energy of the harpoon decreases obviously under the condition of friction, and the harpoon can be successfully embedded in the target plate under the action of friction. Under the condition of not considering the friction coefficient, the harpoon can always leave the target plate as long as the head penetration part flies away from the target plate and a certain amount of kinetic energy remains. The kinetic energy remains almost the same during the stage of contact with the column surface, and the harpoon can always penetrate through the target plate, but it cannot be embedded in the target.

### 3.6. Velocity and Embedding Length

According to the above simulation analysis and combined with the structural characteristics of the harpoon itself, after the head of the harpoon is embedded in the target plate, the rebound phenomenon may occur due to the fluctuation of the target plate, and the anchoring is not reliable after embedding; and, considering the thickness of the target plate and its failure form, the embedding and the anchoring effect of 10 mm at the end of the harpoon is not good, and there may be a risk of it falling off in the process of towing. Therefore, the effective embedding part of the harpoon designed in this paper is the bottom column surface position, and the effective embedding length of the harpoon is 50~90 mm.

**Figure 13:**
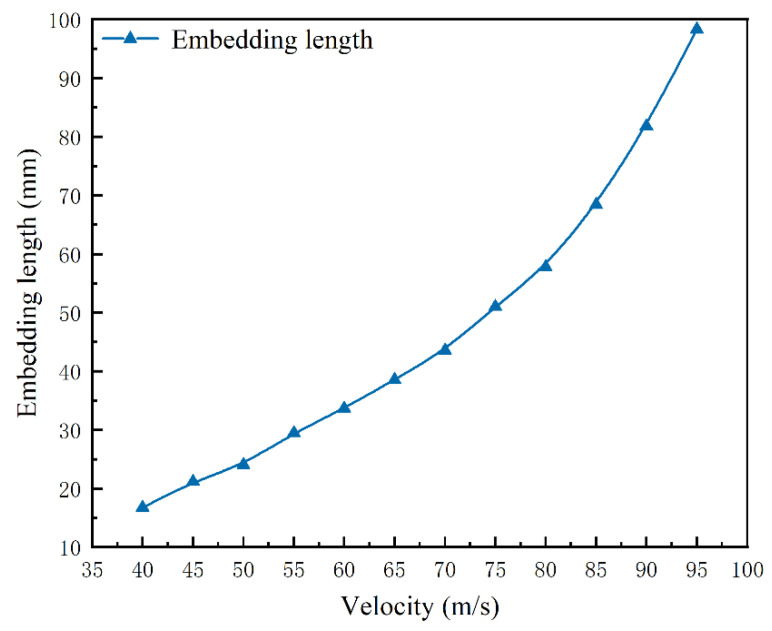
Velocity and embedding length curves.

The impact embedding length at different velocities when tangential contact friction exists between the harpoon and the target plate was studied, and the simulation test design and results are shown in Table 4. The variation curves are shown in Figure 13.

Combined with the relevant data in Table 4 and Figure 13, it can be concluded that when the harpoon velocity is below 70 m/s, it is mainly for the harpoon head to hit the target plate and embed, and the change of the harpoon embedding length at this time is basically linear; when the velocity is greater than 70 m/s, the final form of the embedding form is the contact stage between the harpoon column surface and the target plate, and the offsetting effect of the fluctuation of the target plate by the impact deformation on the velocity of the forward direction of the harpoon is reduced at this stage. The loss of kinetic energy of the harpoon is mainly due to the frictional effect. The embedding length of the harpoon gradually increases with the increase of the velocity, and the increase rate of the embedding length is obvious.

The friction coefficient involved in the above simulations are all fixed at 0.17; however, in the actual problem of harpoon impact embedding, the area of harpoon and target contact contains complex physical and chemical changes, such as hardening, melting, and the phase change of materials. Therefore, the friction coefficient at the time of impact will not be a constant, and it is related to many coefficients, such as the relative velocity of sliding, the pressure on the surface of the harpoon, and the characteristics of the target material [22]. In order to further obtain the suitable interval velocity of the harpoon launch, a simulation of harpoon impact embedding under different friction coefficient conditions was carried out. As the friction coefficient increases, the kinetic energy consumed by the harpoon during the impact embedding process gradually increases, and the required initial launch velocity becomes higher. The simulation test involves the friction coefficient as well as the harpoon embedding length as shown in Table 5.

**Figure 14:**
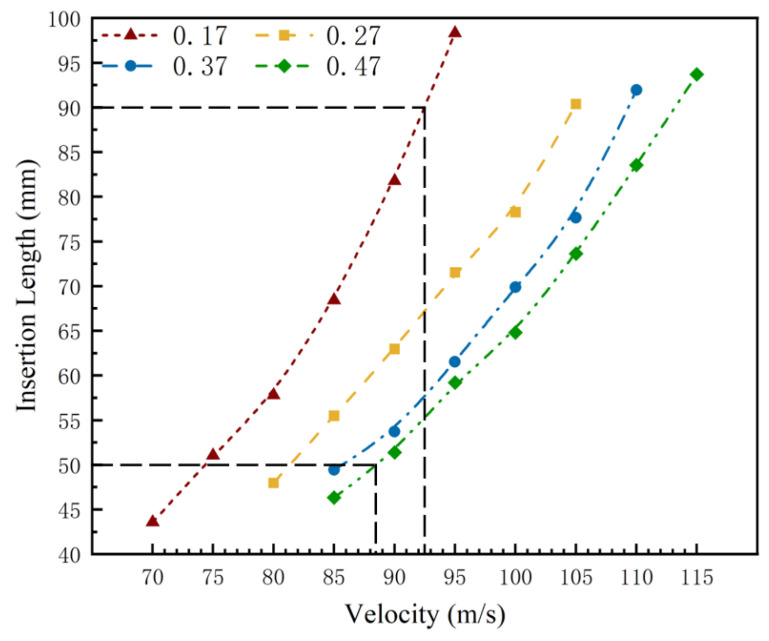
Embedding length and velocity relationship under variable friction coefficient.

Analyzing the above simulation data, the relationship between harpoon embedment length and velocity when the friction coefficient changes is shown in Figure 14. The analysis shows that, on the one hand, as the friction coefficient increases, the velocity required for the harpoon to impact the target plate is higher for the same embedment length; on the other hand, the increase in the friction coefficient leads to a gradual increase in the length range of the harpoon embedded in the target plate, and a larger friction coefficient is beneficial for the harpoon to be embedded in the target plate. In order to accommodate the effective embedding length range of the harpoon within 50~90 mm for all friction coefficients, the initial velocity of the harpoon at launch needs to be controlled in the range of 88.4~92.5 m/s.

## 4. Conclusions

In this paper, the influence of the law of friction coefficient in the low-velocity impact process is studied from the kinetic energy change and embedding length of the harpoon in the impact process through the impact test, combined with the simulation numerical simulation. The main conclusions are obtained as follows:

1. In the problem of the harpoon impacting and embedding in the target plate at a velocity lower than the ballistic limit, the tangential friction effect is more obvious for the consumption of the kinetic energy of the harpoon itself, and it cannot be ignored;
2. When the head of the harpoon is embedded in the target plate, due to which the fluctuation of the target plate may rebound after the impact of the head of the harpoon, the embedding is not reliable. The effective embedding part of the harpoon is the bottom column surface position. Considering the influence of the target plate deformation on embedding, the effective embedding length range of the harpoon is 50~90 mm;
3. Considering the effect of friction coefficient change, in order to adapt to the effective embedding length range of the harpoon under each friction coefficient condition to within 50~90 mm, the theoretical launch initial velocity of the harpoon should be between 88.4~92.5 m/s.

## Figures and Tables

**Table 1:** Harpoon and target plate material parameters.

Model	Density/ g·cm^−3^	Elastic Modulus/ Gpa	Poisson Ratio	Johnson-Cook Model Parameters				
				*A*/MPa	*B*/Mpa	*n*	*C*	*m*
Harpoon	7.85	210	0.33	714	563	0.518	0.064	0.698
Target plate	2.77	71.7	0.33	375	592	0.42	0.001	1.426

**Table 2:** Harpoon embedding experiment.

No.	Velocity/m·s^−1^	Experimental Embedding Length/mm	Simulation Embedding Length/mm
1	50.7	22	23.1
2	65	37	38.58
3	76.4	51.5	51.03

**Table 3:** Simulation experimental design and results.

No.	Friction Coefficient	Velocity/m·s^−1^	Embedding Length—With Friction/mm	Embedding Length—Without Friction/mm
1	0.17	37.5	15.48	25.71
2	0.17	40	16.74	29.41
3	0.17	42.5	19.37	33.99
4	0.17	45	21.18	36.63

**Table 4:** Simulation tests and results in the presence of tangential contact friction.

No.	Friction Coefficient	Velocity/m·s^−1^	Embedding Length/mm
1	0.17	40	16.74
2	0.17	45	21.18
3	0.17	50	24.05
4	0.17	55	29.49
5	0.17	60	33.67
6	0.17	65	38.58
7	0.17	70	43.57
8	0.17	75	51.03
9	0.17	80	57.80
10	0.17	85	68.42
11	0.17	90	81.78
12	0.17	95	98.32

**Table 5:** Harpoon embedding length.

No.	Friction Coefficient	Velocity/m·s^−1^	Embedding Length/mm
1	0.17	70	43.57
		75	51.03
		80	57.80
		85	68.42
		90	81.78
		95	98.32
2	0.27	80	47.96
		85	55.48
		90	62.96
		95	71.52
		100	78.27
		105	90.37
3	0.37	85	49.42
		90	53.72
		95	61.51
		100	69.88
		105	77.65
		110	91.94
4	0.47	85	46.31
		90	51.37
		95	59.19
		100	64.78
		105	73.61
		110	83.54
		115	93.68

## Data Availability

Not applicable.

## References

[1] Wei Z., Zhang H., Zhao B., Liu X., Ma R. (2020). Impact Force Identification of the Variable Pressure Flexible Impact End-Effector in Space Debris Active Detumbling. Appl. Sci..

[2] Adushkin V.V., Aksenov OYu Veniaminov S.S., Kozlov S.I., Tyurenkova V.V. (2020). The small orbital debris population and its impact on space activities and ecological safety. Acta Astronaut..

[3] Ru M., Zhan Y., Cheng B., Zhang Y. (2022). Capture Dynamics and Control of a Flexible Net for Space Debris Removal. Aerospace.

[4] Virgili B.B., Krag H. Analyzing the criteria for a stable environment. Proceedings of the AAS/AIAA Astrodynamics Specialist Conference.

[5] Liou J.-C., Johnson N., Hill N. (2010). Controlling the growth of future LEO debris populations with active debris removal. Acta Astronaut..

[6] Forshaw J.L., Aglietti G.S., Fellowes S., Salmon T., Retat I., Hall A., Chabot T., Pisseloup A., Tye D., Bernal C. (2019). The active space debris removal mission RemoveDebris. Part 1: From concept to launch. Acta Astronaut..

[7] Reed J., Barraclough S. (2013). Development of Harpoon System for Capturing Space Debris. Eur. Conf. Space Debris.

[8] Campbell J., Hughes K., Vignjevic R., Djordjevic N., Taylor N., Jardine A. (2022). Development of modelling design tool for harpoon for active space debris removal. Int. J. Impact Eng..

[9] Dudziak R., Tuttle S., Barraclough S. (2015). Harpoon technology development for the active removal of space debris. Adv. Space Res..

[10] Aglietti G.S., Taylor B., Fellowes S., Salmon T., Retat I., Hall A., Chabot T., Pisseloup A., Cox C., Zarkesh A. (2019). The active space debris removal mission RemoveDebris. Part 2: In orbit operations. Acta Astronaut..

[11] Fras T., Roth C.C., Mohr D. (2019). Dynamic perforation of ultra-hard high-strength armor steel: Impact experiments and modeling. Int. J. Impact Eng..

[12] Wang Y., Chen X., Xiao X., Vershinin V.V., Ge R., Li D.-S. (2020). Effect of Lode angle incorporation into a fracture criterion in predicting the ballistic resistance of 2024-T351 aluminum alloy plates struck by cylindrical projectiles with different nose shapes. Int. J. Impact Eng..

[13] Kpenyigba K., Jankowiak T., Rusinek A., Pesci R., Wang B. (2015). Effect of projectile nose shape on ballistic resistance of interstitial-free steel sheets. Int. J. Impact Eng..

[14] Deng Y., Wu H., Zhang Y., Huang X., Xiao X., Lv Y. (2022). Experimental and numerical study on the ballistic resistance of 6061-T651 aluminum alloy thin plates struck by different nose shapes of projectiles. Int. J. Impact Eng..

[15] Rusinek A., Rodríguez-Martínez J., Arias A., Klepaczko J., López-Puente J. (2008). Influence of conical projectile diameter on perpendicular impact of thin steel plate. Eng. Fract. Mech..

[16] Johnson G.R., Cook W.H. A constitutive model and data for metals subjected to large strains, high strain rates and high tem-peratures. Proceedings of the 7th International Symposium on Ballistics.

[17] Elek P.M., Jaramaz S.S., Micković D.M., Miloradović N.M. (2016). Experimental and numerical investigation of perforation of thin steel plates by de-formable steel penetrators. Thin-Walled Struct..

[18] Li X., Li J., Zhao Z., Liu D., Ou-Yang X. (2008). Numerical study on penetration of a high-speed-rotating bullet into the moving sheet-metal plate. Impact Dyn..

[19] Gang W., Wei Z., Yunfei D. (2019). Identification and validation of constitutive parameters of 45 Steel based on J-C model. J. Vib. Shock.

[20] Chen J., Xu W., Xie R., Zhang F., Hu W., Huang X., Chen G. (2017). Sample size effect on the dynamic torsional behaviour of the 2A12 aluminium alloy. Theor. Appl. Mech. Lett..

[21] Rusinek A., Rodríguez-Martínez J., Zaera R., Klepaczko J., Arias A., Sauvelet C. (2009). Experimental and numerical study on the perforation process of mild steel sheets subjected to perpendicular impact by hemispherical projectiles. Int. J. Impact Eng..

[22] Li J., Zhang X., Liu C., Chen H., Wang J., Xiong W. (2021). Study on mass erosion model of projectile penetrating concrete at high speed considering variation of friction coefficient. Explos. Shock. Waves.

